# Supporting Workers to Sit Less and Move More Through the Web-Based BeUpstanding Program: Protocol for a Single-Arm, Repeated Measures Implementation Study

**DOI:** 10.2196/15756

**Published:** 2020-05-04

**Authors:** Genevieve Nissa Healy, Ana D Goode, Alison Abbott, Jennifer Burzic, Bronwyn K Clark, David W Dunstan, Elizabeth G Eakin, Matthew Frith, Nicholas D Gilson, Lan Gao, Lynn Gunning, Jodie Jetann, Anthony D LaMontagne, Sheleigh P Lawler, Marjory Moodie, Phuong Nguyen, Neville Owen, Leon Straker, Perri Timmins, Lisa Ulyate, Elisabeth A H Winkler

**Affiliations:** 1 School of Public Health The University of Queensland Brisbane Australia; 2 Baker Heart and Diabetes Institute Melbourne Australia; 3 Curtin University Perth Australia; 4 Workplace Health and Safety Queensland Office of Industrial Relations Queensland Australia; 5 Mary MacKillop Institute for Health Research Australian Catholic University Melbourne Australia; 6 Kin8 Melbourne Australia; 7 School of Human Movement and Nutrition Science Brisbane Australia; 8 School of Health & Soc. Dev, Deakin University Melbourne Australia; 9 The University of Newcastle Callaghan Australia; 10 Comcare Canberra Australia; 11 Institute for Health Transformation, Deakin University Geelong Australia; 12 Swinburne University of Technology Melbourne Australia; 13 Safe Work Australia Canberra Australia

**Keywords:** implementation trial, workplace, sitting, health promotion, activity, health and safety, public health, occupational, evaluation, web-based

## Abstract

**Background:**

The web-based BeUpstanding Champion Toolkit was developed to support work teams in addressing the emergent work health and safety issue of excessive sitting. It provides a step-by-step guide and associated resources that equip a workplace representative—the champion—to adopt and deliver the 8-week intervention program (BeUpstanding) to their work team. The evidence-informed program is designed to raise awareness of the benefits of sitting less and moving more, build a supportive culture for change, and encourage staff to take action to achieve this change. Work teams collectively choose the strategies they want to implement and promote to stand up, sit less, and move more, with this bespoke and participative approach ensuring the strategies are aligned with the team’s needs and existing culture. BeUpstanding has been iteratively developed and optimized through a multiphase process to ensure that it is fit for purpose for wide-scale implementation.

**Objective:**

The study aimed to describe the current version of BeUpstanding, and the methods and protocol for a national implementation trial.

**Methods:**

The trial will be conducted in collaboration with five Australian workplace health and safety policy and practice partners. Desk-based work teams from a variety of industries will be recruited from across Australia via partner-led referral pathways. Recruitment will target sectors (small business, rural or regional, call center, blue collar, and government) that are of priority to the policy and practice partners. A minimum of 50 work teams will be recruited per priority sector with a minimum of 10,000 employees exposed to the program. A single-arm, repeated-measures design will assess the short-term (end of program) and long-term (9 months postprogram) impacts. Data will be collected on the web via surveys and toolkit analytics and by the research team via telephone calls with champions. The Reach, Effectiveness, Adoption, Implementation, and Maintenance Framework will guide the evaluation, with assessment of the adoption/reach of the program (the number and characteristics of work teams and participating staff), program implementation (completion by the champion of core program components), effectiveness (on workplace sitting, standing, and moving), and maintenance (sustainability of changes). There will be an economic evaluation of the costs and outcomes of scaling up to national implementation, including intervention affordability and sustainability.

**Results:**

The study received funding in June 2018 and the original protocol was approved by institutional review board on January 9, 2017, with national implementation trial consent and protocol amendment approved March 12, 2019. The trial started on June 12, 2019, with 48 teams recruited as of December 2019.

**Conclusions:**

The implementation and multimethod evaluation of BeUpstanding will provide the practice-based evidence needed for informing the potential broader dissemination of the program.

**Trial Registration:**

Australian New Zealand Clinical Trials Registry ACTRN12617000682347; https://www.anzctr.org.au/Trial/Registration/TrialReview.aspx?id=372843&isReview=true.

**International Registered Report Identifier (IRRID):**

DERR1-10.2196/15756

## Introduction

### Background

A growing body of recent evidence links high volumes of sitting time to risk of major chronic diseases and premature mortality [1]. Only very high volumes of moderate- to vigorous-intensity physical activity (≥60 min per day), which are achieved by less than 5% of the population, have been seen to attenuate the risk of death associated with high sitting time, according to a recent meta-analysis using data from over 1 million adults [2]. Correspondingly, the national physical activity and health guidelines have a dual message of move more *and* sit less [3].

Sitting time can be strongly contextually driven, dictated by the environmental and social settings in which it occurs [4]. For many working adults, the majority of daily sitting time is accrued in the occupational environment [5], with desk workers spending on average 70% to 80% of their working day sitting [6]. Much of this sitting time is accrued in prolonged, unbroken bouts of 30 min or longer [6]: a pattern that potentially places them at increased risk for poor cardiometabolic [7,8] and musculoskeletal [9] health. As the proportion of industry sectors that involve desk-based work has increased substantially in recent decades, with further increases being forecast [10], the desk-based workplace has been identified as a key setting in which to target reductions in prolonged sitting time [11]. The relevance for occupational health and safety, as well as for public health, of addressing this behavior is reflected in Safe Work Australia’s acknowledgment of prolonged workplace sitting as an emergent work health and safety issue [12].

Within this context, the Stand Up Australia collaborative research program was developed [13]. Its aim was to understand how to reduce prolonged sitting time in the workplace and the benefits that may ensue, with the explicit intention of informing translation into practice. A series of pragmatic, researcher-led intervention trials, with participant numbers ranging from 32 to 231, assessed the effectiveness of different strategies (organizational, and environmental, individual; alone or in combination) to support workers to stand up, sit less, and move more in the workplace, with a particular focus on the desk-based workplace [6,14-18]. This Stand Up Australia program of research demonstrated that it is feasible and acceptable to introduce strategies within desk-based workplaces to create a dynamic work environment (which encourages more movement, more often) and to do so without detrimentally impacting on productivity [19]. Such strategies can lead to reductions in workplace sitting time that are substantial (eg, >1.5 hours per 8 hours at the workplace [14]) and sustained (≥12 months [6]). These findings have further been corroborated by other research groups [20,21] and supported by several systematic reviews [22-26]. With a body of evidence on the feasibility and benefits of reducing workplace sitting time, there is now a strong demand for advice, assistance, and support in implementing evidence-based strategies into policy and practice. However, tools and resources to support such implementation at scale do not exist. To meet this appetite, the BeUpstanding Champion Toolkit was developed collaboratively based on evidence from Stand Up Australia and the broader sedentary behavior and health research field.

The no-cost, web-based BeUpstanding Champion Toolkit [27] provides a step-by-step implementation guide and associated multimedia resources to enable a workplace champion to deliver the intervention program (BeUpstanding) within their own work team, independent of input from external expert stakeholders (ie, researchers) [13]. In line with better practice [28] and existing frameworks for program delivery [29], the program is underpinned by a participative and collaborative approach, tailoring of strategies to the organization, visible organizational support for the program, a strong evaluation framework, and communication of program outcomes, including through automated reports. The program allows for repeated delivery, with champions encouraged to continue to make sustainable changes and build on previous success within their work teams. However, in a key distinction from the researcher-led Stand Up Australia interventions, BeUpstanding was designed specifically for delivery by workplace champions (ie, dedicated staff members). A *train-the-champion* approach was used as workplace champions have been shown to be critical to the success of workplace interventions, acting as role models and drivers for staff participation and work team change [30-32]. This approach also facilitates wide-scale delivery as the workplace (rather than the research team) is responsible for program delivery.

The translation of what has been learned from the Stand Up Australia intervention trials to the BeUpstanding program has involved multiple, iterative phases [13]. These phases have been underpinned by the key principles guiding dissemination of broad-reach health behavior programs [33], including partnerships with key stakeholders, ensuring fit of the program with the organizational goals, integration of outcomes important to informing funders and advancing science, systematic tracking of the resources needed for implementation and intervention, and the maintenance of program fidelity while being flexible and responsive. Central to this has been the development of the technology platform underpinning the toolkit. This platform has not only enabled the evaluation of the effectiveness of the program but has also facilitated insights into the levels of engagement with the program components.

Phase 1, described in detail elsewhere [13], involved initially creating BeUpstanding from the Stand Up Australia interventions. This development occurred in close collaboration with government occupational health, safety, and well-being partners to ensure strong alignment with existing workplace health, safety, and wellness frameworks. It was also developed with consideration of the partner requirements (optimization criteria [34]) that the program have the following attributes: low cost or no cost to workplaces, feasible for workplaces to deliver, scalable, and compatible with existing programs, including the frameworks and language used. These considerations, and the learnings from the preceding trials, collectively led to the “train-the-champion” approach, the use of a web-based toolkit, and the framing of the intervention around the three stages commonly used in government workplace health, safety, and well-being programs (ie, *Plan, Do,* and *Review*). The low cost/no cost requirement also meant that sit-stand workstations, which have been shown to effectively reduce workplace sitting (particularly when part of a multicomponent approach) [35], are not a core component or requirement for participation in the program.

Phase 2 involved a quantitative and qualitative evaluation of a small-scale pilot of the beta (test) version of the toolkit [36]. Seven teams of workers in mostly desk-based occupations were included, collectively covering diverse sectors: blue and white collar sectors; government and nongovernment; metropolitan and regional; and small, medium, and large organizations. Overall, the pilot phase demonstrated that the BeUpstanding Champion Toolkit (beta version) was feasible and acceptable for use by workplace champions and that the program delivered through the toolkit was effective at raising awareness, building a supportive work team culture, and reducing workplace sitting time [36,37]. The piloting of the toolkit showed an average reduction in self-reported workplace sitting time of 34 min per 8-hour workday (95% CI −51 min to −14 min) following approximately 3 months of intervention. This level of effect on sitting time has previously demonstrated significant improvements in some indicators of cardiometabolic health [38]. Champions typically spent 30 min to 1 hour per week on the program during this pilot phase [36]. Notably, interviews with the workplace champions 12 months after initial implementation found that teams continued to support the strategies, including through policy development (eg, centralized printers) and dedicated resource funding (eg, purchase of sit-stand desks) [37].

The learnings from phase 2 then informed the optimization of the toolkit (phase 3) to ensure it was fit for purpose for an implementation trial. Phase 3 included the development of a web-based, user-friendly onboarding system (to both promote the toolkit and enable champions to sign up for the toolkit) using human-centered design principles [39], enhanced backend capacity of the toolkit (to facilitate multiple simultaneous users), development of an embedded survey management and data collection system, and enhanced graphic design.

This updated version was tested via a *soft launch* of the program, with over 100 champions enrolling in the program during this period (September 2017 to May 2019). Several key learnings were gained from these early adopters. Firstly, despite the minimal promotion during the soft launch, there was strong uptake of the program, with champions enrolled from throughout Australia and across multiple sectors. This provides strong indication that there is an industry need for a program such as BeUpstanding. Secondly, workplaces were at different stages of readiness, with some champions wanting only to use select program materials (eg, posters) to help raise awareness of the importance of sitting less and moving more, whereas others were ready to run the full program. Thirdly, there was wide variation in how champions engaged with the toolkit, measured by the number of log-ons, with some champions repeatedly logging on throughout the program and others logging on rarely and/or infrequently. Finally, we found that although the toolkit was designed well for delivery by a single champion to their team of workers, it was not sufficiently flexible for larger organizations with large workplaces. It was identified that in a number of instances, there was a combined team formed of several teams led by champions who each adopted more nuanced roles (such as oversight without necessarily directly intervening on staff). Adaptations to the toolkit were made accordingly to suit a range of toolkit user roles.

These key learnings, which were complemented by discovery interviews and in-depth case studies with select participants (chosen to capture insights across sectors, locations, organizational size, and toolkit engagement), were used to inform further optimization of the program and toolkit and the protocol development for the national implementation trial of the BeUpstanding program (phase 4). Adaptions were done taking into account considerations from multiple perspectives, including the end users, the partners, the researchers, and the financial constraints [34,40]. The aims of this paper are to describe the current version of the BeUpstanding program and the methods and protocol for evaluating the BeUpstanding program in the context of a national implementation trial.

### BeUpstanding Program

The BeUpstanding program is designed to be implemented within a workplace (broadly, defined as from one organization, with the same workplace policies) by a champion to their work team (colocated members of the workplace) of which the champion is also a member. Larger workplaces may run BeUpstanding by having several champions deliver the intervention to their teams concurrently. For the purposes of accrual targets and statistical analyses, these multiple teams are counted as one combined *team*. There are three phases to the program (plan, do, and review) and five steps as part of the BeUpstanding program (Table 1). Each step has associated tasks for the champion to complete, noting that not all tasks may be relevant for all champions because of their workplace and/or work team requirements. The toolkit provides information (*training*) on the purpose of each step and task and resources to support the implementation of each task. As part of the implementation trial, champions will receive further training via coaching calls. The most critical step of the program is the staff workshop (Step 3.3). This step is designed to get everyone in the work team on board in terms of why and how the team can BeUpstanding together. In line with participatory design principles [41], work teams are encouraged to collectively choose three strategies to stand up, sit less, and move more to implement, based on which best suit their team’s needs and existing culture. Some strategy suggestions, according to the hierarchy of control [42], are provided within the toolkit (Table 2 shows a modified version of this resource). Staff members may choose to implement more than the three team strategies. Alternate suggestions for raising awareness and enabling this collective decision making are provided when running the workshop with all staff at the same time is infeasible (eg, because of shift work). Champions are encouraged to run the BeUpstanding program for 8 weeks from the launch, sending emails and rotating posters on a weekly basis for the first 4 weeks and fortnightly for the second 4 weeks, with the posters and emails organized according to the recommended schedule. Collectively, the workshop, posters, and emails are designed to raise awareness of the benefits of sitting less and moving more, build a supportive culture for change, and encourage participants to take action to achieve this change. Owing to the participative nature of choosing the strategies, and the ability of the champion to tailor the emails, the actual intervention program is bespoke for each work team. The champion is responsible for running and evaluating the program, which includes sending all staff in their work team links to the web-based evaluation surveys (Task 2.2; Task 5.1). Champions are also encouraged to hold staff events (eg, a lunchtime walk and wear your sneakers to work day) and to celebrate and promote individual and whole-of-team success. All staff in the work team will potentially be exposed to the intervention messages (posters and emails), and all staff can choose their level of involvement with both the strategies and the evaluation components. The toolkit encourages champions to run BeUpstanding (or components of thereof) with their team on an annual basis.

**Table 1:** Phases, steps, champion tasks, supporting resources and rationale for the steps of the BeUpstanding Champion Toolkit.

Phase and steps		Champion tasks		Supporting resources		Rationale of step	
**Plan,** **approximately 1-2 months (variable)**							
	Step 1: Getting support from management		Make a case for BeUpstanding Formalize management’s commitment in writing		Business case template Sample policy Journey map		To build the business case for running the program and formalize management commitment (if required)
	Step 2: Needs assessment		Conduct a workplace audit^a^ Conduct a staff survey^a^		Staff email templates and posters Links to workplace audit and staff survey Audit report and links to staff survey results		To help the champion: assess their current workplace environment and existing policies and identify available resources and facilities and opportunities to support staff to stand up, sit less and move more. To assess the need for BeUpstanding and provide a baseline to be able to measure any changes arising from the program in terms of staff behaviors, attitudes, beliefs, and health, productivity, and well-being indicators.
	Step 3: Preparing for the program		Create and maintain a support network Hold a well-being committee workshop Hold a staff consultation workshop^a^ Promote BeUpstanding strategies^a^		Well-being committee member invitation template/video/staff consultation planning tool BeUpstanding PowerPoint presentation for staff workshop BeUpstanding staff information video Strategy survey and associated poster generation		The well-being committee (recommended 3-6 members, mix of management and general staff, and fortnightly meetings) is intended to provide support to the champion in implementing the BeUpstanding program. The staff consultation workshop (or equivalent) is designed to create ownership of the program and strategies by the workteam and ensure everyone has the same base level of knowledge regarding the benefits of sitting less and moving more. The web-based strategy survey enables data collection of the team strategies chosen and promotional support for these strategies via the generation of a customized poster.
**Do, approximately 8 weeks**							
	Step 4: Putting it into practice		Set an action plan and launch Promote with posters and health information^a^ Promote with email reminders to staff^a^ Encourage change champions, and celebrate success		Action plan example and template BeUpstanding posters No/low-cost tips and tools Recommended emails and additional email guide/templates Change champion guide		To support champions to put their BeUpstanding strategies into practice through highlighting key activities and people involved, resource requirements, and the program timeline including evaluation tasks and tools. To raise awareness, build culture, and encourage action around standing up, sitting less, and moving more.
**Review, approximately 1 month**							
	Step 5: Evaluation		Do follow-up staff survey^a^ Do program completion survey^a^ Where to from here		Links to follow-up surveys and staff survey results Team performance report and completion certificate		To support the champion and the work team to evaluate and reflect on their progress and plan for sustainability.

^a^Steps marked as critical within the toolkit (core components).

**Table 2:** Suggested team-level strategies to BeUpstanding according to the Hierarchy of Control (adapted from Resource 3.2).

Hierarchy of control	Strategies
Elimination	Use technology (eg, voice recognition software) to eliminate prolonged sedentary tasks
Substitution (redesign)	Enable internal stair access and workplace re-design to facilitate more movement where possible Move water, bins, and printers away from desks Install height-adjustable workstations Provide designated standing areas (eg, in tea rooms and meetings rooms) Provide facilities such as showers and lockers to encourage active transport and physical activity Use phone support accessories (eg, headphones and speaker phones) to facilitate standing during phone-based tasks
Administration	Create a walking track around workplace Encourage workers to leave desks during breaks Provide organizational support for flexible hours for lunch breaks to encourage physical activity (eg, gym visits) Encourage face-to-face interaction with colleagues Stand up and move around when taking a phone call (where possible) Undertake walking meetings Conduct standing meetings Encourage staff to regularly walk to top up water glass/bottle Use signage (eg, posters) to support BeUpstanding messages Use computer software to prompt breaks from sitting Provide physical prompts at desk to stand regularly (eg, stickers) Leave desk in standing position when leaving workspace (if using height-adjustable workstations) Conduct daily group activity sessions Undertake a team challenge (eg, 10,000 steps challenge)

#### BeUpstanding Intervention Messages and Behavioral Targets

The program’s behavioral targets are to achieve an even 50:50 split between sitting and nonsitting (ie, upright) activities at work and to alternate posture at least every 30 min between sitting and upright (or vice versa)—consistent with public, occupational, and clinical guidelines [43-45]. To support these targets, the BeUpstanding intervention messages are to *Stand Up, Sit Less, Move More*. *Stand Up* is a prompt to break up long periods of sitting, *Sit Less* is a prompt to reduce overall sitting time throughout the day by swapping some sitting with either standing or moving, and *Move More* is a prompt to increase physical activity (primarily opportunistic, incidental activity) throughout the day. Increased activity and decreased sitting are primarily targeted through organizational, environmental, and social approaches. Messaging throughout the resources encourages regular postural shifts and reminders to *listen to your body* in recognition that there are also adverse outcomes associated with prolonged unbroken standing [46-48]. No specific individual-level support for staff is provided through the toolkit.

#### BeUpstanding Website

The BeUpstanding program is delivered via the BeUpstanding Champion Toolkit hosted on the BeUpstanding website [27]. The website is hosted, maintained, and updated by project staff, with all data stored in a secure, cloud-based system (Microsoft Azure) that is backed up weekly to the University of Queensland servers (lead investigator’s team: GH, AG, JB, JJ, LU, EW). The toolkit itself is powered through a bespoke platform that includes in-built systems that facilitate survey design, project management, and user tracking, enabling the research team to readily track a champion’s progress and engagement through the program and collect survey-based data. In addition to the toolkit, the BeUpstanding website (freely available) also includes pages on the business case and associated promotional materials for running the BeUpstanding program, the evidence base supporting the BeUpstanding program, a checklist to ensure program readiness, a link to the BeUpstanding blog and social media, a frequently asked questions section, and details on the investigators and partners. Champions are encouraged to visit the blog via monthly electronic newsletters for the latest research evidence and tips for running the program.

## Methods

### Aims and Research Questions

The aim of this study is to evaluate the BeUpstanding program in the context of a national implementation trial. The research questions to be answered are those important to informing the dissemination (phase 5) [13]: in particular, who takes part in the program, how the program was delivered, did the program work (and for whom did it not work), and how much did it cost. The RE-AIM (Reach, Effectiveness, Adoption, Implementation, and Maintenance) Framework [49] will be used to guide the evaluation, with assessment of the *adoption/reach* of the program (the number and characteristics of work teams and participating staff), program *implementation* (completion by the champion of core program components), *effectiveness* (on workplace sitting, standing, and moving), and *maintenance* (sustainability of changes). The implementation trial is funded by a National Health and Medical Research Council (NHMRC) of Australia Partnership Project Grant (number 1149936), which includes cash and/or in kind support from the five partners (see below). Ethical approval was gained by The University of Queensland Human Research Ethics Committee (approval number 2016001743). The trial was prospectively registered on May 12, 2017 (ACTRN12617000682347), before the soft launch of the program and last updated on the June 11, 2019. All participants will provide informed consent to participate.

### Study Design

A single-arm design will be used to evaluate the BeUpstanding program, with repeated cross-sectional evaluations at preprogram (0 weeks), end of program (approximately 8 weeks; primary endpoint), and at 9 months postprogram (approximately 12 months post sign up). Repeated cross-sectional evaluations provide a flexible evaluation protocol [50] that can assess change within retained members of the baseline survey cohort over time and more general time trends (owing to both changes over time within participants and some fluidity in work team membership, such as because of workforce turnover).

### Study Eligibility and Accrual Targets

On the basis of data reported by the champion as part of the web-based registration process, eligible Australian-based work teams will be those who had not run the BeUpstanding program previously with a minimum of five staff members, job roles or tasks that predominantly involve desk-based work, and a staff member willing to perform the duties of a workplace champion. Champions must also be planning to run the program within the recruitment window. For large organizations, including those located across numerous sites, multiple work teams from the one organization will be eligible to participate. These will be treated as a single combined *team* when the intervention is concurrent and within a workplace as per the criteria; otherwise, separate teams will be permitted to participate. Each champion will invite all employees within their work team to participate in the program and its evaluation. All workers invited will be considered eligible unless they indicate within the staff survey that they are unable to currently walk or stand for at least 10 min without an assistive device or requiring assistance from another person. Accrual targets have been set at 50 or more work teams per priority sector and 10,000 or more staff exposed to the program in total (see sample size). Performance against these accrual targets will be reviewed at the quarterly steering committee meetings, with the promotion and marketing plan adapted as required to ensure targets are met.

### Study Partners and Promotion

The implementation trial will be conducted in partnership with five Australian workplace health and safety policy and practice organizations: Safe Work Australia, Comcare, Queensland Office of Industrial Relations, The Victorian Health Promotion Foundation (VicHealth), and Healthier Workplace Western Australia. These organizations are responsible for developing, implementing, and/or promoting Australian workplace health and safety policy. Each partner has committed to endorse and promote the toolkit across their relative jurisdictions. Desk-based employees from a wide cross-section of industries will be targeted, inclusive of sectors collectively identified as priorities by the partners (small business, regional, call center, blue collar, and government). To ensure efforts are coordinated, a detailed action-mobilization plan will be developed with the partners. The plan, which will include an annual promotional *push* via an awareness raising event, will build on and coordinate with existing communication channels and resources from the partners and participating institutes, including social media, web links, email listservers, newsletters, workplace health promotion and occupational health networks, conferences, and workshops.

### Study Protocol for the Implementation Trial

The BeUpstanding website [27] is designed for workplace champions; however, anyone can freely sign up to use the BeUpstanding Champion Toolkit via the registration survey (sign up form) on the BeUpstanding website. At signup, a user identifier is generated and a welcome email is automatically sent that includes details regarding the implementation trial. To unlock the toolkit contents, the user is required to complete the champion profile survey and is asked to nominate their intended role as a toolkit user (which might be a workplace champion or another nondelivery role, such as senior decision maker, interested staff member.). Following completion of this survey, champions with work teams that appear eligible for the implementation trial will be invited via a phone call from the research team to participate in the implementation trial, with recruitment continuing until accrual targets are met. This phone call with the champion will be used to confirm the eligibility of the work team for involvement in the implementation trial, ascertain from the champion the likely readiness of the work team to participate in the program, and confirm the contact details of the workplace champion (and an alternate contact). Those eligible and indicating interest in trial participation will be sent additional information on trial participation requirements, namely, confirmation of organizational support to run the five-step BeUpstanding program and commitment to the implementation trial evaluation components. The champion’s electronic consent to the trial will be required before implementation trial enrolment.

### Data Collection

**Figure 1:**
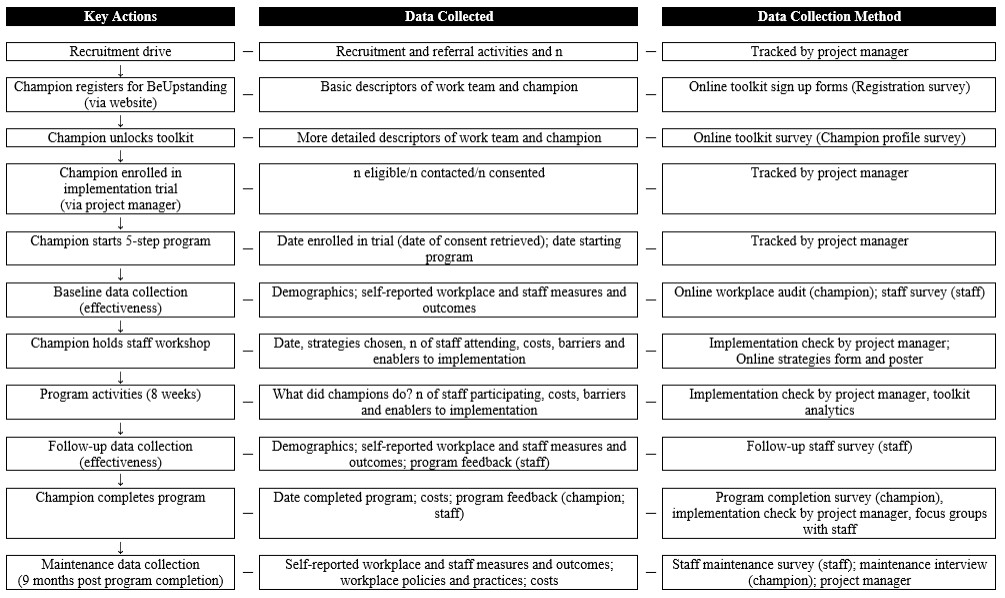
Key actions, data collected, and data collection method of the BeUpstanding implementation trial. Staff focus groups will be conducted in a sub-sample of teams only; separate consent will be sought from staff members for participation in this component.

Outcome and process data and the characteristics of the workplaces, champions, and staff taking part in the implementation trial will be collected via the dedicated, stand-alone BeUpstanding website (Registration Survey; Champion Profile Survey; Workplace Audit; Staff Surveys —baseline, end program, and maintenance; Strategy Survey; Program Completion Survey; and toolkit analytics) and by the project manager (implementation checks and qualitative interviews), as outlined in Figure 1. Champions will be required to provide informed web-based consent for their data to be used by the research team before completing the Champion profile survey, with further consent required to participate in the implementation trial. Staff will be required to provide informed consent for their data to be used by the research team before completing each of the staff surveys. Data for staff are anonymous; however, to enable participants to be tracked across data collection points, each staff survey includes three questions designed to generate a unique (but anonymous) identifier for the staff participant when used in combination with the champion ID: day of the month they were born on, first letter of mothers first name, and last three digits of their mobile number.

The promotional activities undertaken by partners will be recorded at the 6-weekly partner meetings, with their impact on registrations tracked through the analytics in the toolkit website. The promotional pathways will be tracked through URL identifiers, through Google Analytics, and via champion self-reporting through the champion profile survey. Factors potentially influencing uptake and engagement with the program (eg, number of teams within a workplace participating in the program) will also be tracked via the registration survey and implementation checks. To ensure minimum data accrual targets are met, the project manager will follow up with champions (via email/phone) where necessary to encourage and support data collection.

The project manager will have a minimum of five telephone contacts with the champion across the implementation trial evaluation: (1) recruitment, (2) confirmation of consent and explanation of next steps, (3) as soon as possible following the staff workshop, (4) at the end of the program, and (5) 9 months after the end of the program. Focus groups will be undertaken with a subsample of consenting staff from participating teams (n was approximately 15) at the end of the program to assess their perspectives on the processes and outcomes of the program. A mix of teams who made small/no, midrange, and large improvements and from different sectors will be purposively sampled, with focus groups conducted either in person or on the web via a virtual meeting room.

### Outcomes and Measures

Outcomes and measures are shown in Table 3, along with the relevant RE-AIM indicators and measurement tools. As adoption logistically occurs before reach, RE-AIM is reported as ARIEM.

**Table 3:** Outcomes, measures, and assessment tools of the BeUpstanding implementation trial according to the Reach, Effectiveness, Adoption, Implementation, and Maintenance framework.

Reach, Effectiveness, Adoption, Implementation, and Maintenance dimensions		Collection method/assessment tools
**Adoption by teams**		
	Champions registering for BeUpstanding (n)	Registration (sign up) survey
	Champions unlocking the toolkit (n)	Champion profile survey
	Characteristics of champions and their organizations and their work teams (including size of organization and number of staff)	Champion sign on, Champion profile survey; workplace audit
	Reasons for taking up the program	Champion profile survey
	Champions eligible and enrolling in implementation trial, n (%) of eligible	Champion profile survey
	Champion withdrawals from implementation trial (n) and reasons for withdrawal	Implementation check
**Reach of Staff in Teams**		
	Staff in work team (n as reported by champion)	Champion profile survey; implementation check
	Percentage of staff in work team that participate in choosing BeUpstanding strategies	Strategy survey; implementation check
	Participation in staff surveys, n (%)	Staff surveys (champion-reported n for %)
	Characteristics of staff taking part in the evaluation	Staff surveys
**Implementation**		
	Completion rates	Toolkit analytics; implementation check
	Engagement with the program	Toolkit analytics, implementation check; program completion survey
	Strategies chosen by work team	Strategy survey; implementation check
	Sit less, move more strategies (staff)	Staff surveys
	Barriers and enablers to implementation	Implementation check
**Effectiveness**		
	Workplace sitting and activity	Staff surveys
	Activity preference alignment	Staff surveys
	Organizational social norms	Staff surveys
	Enablers to sitting less and moving more	Staff surveys; staff focus groups^a^
	Perceived barriers to sitting less and moving more	Staff surveys; staff focus groups^a^
	Work performance and engagement	Staff surveys
	General health	Staff surveys
	Adverse/unintended consequences (end program only) for champions and staff	Implementation check; staff follow-up survey; program completion survey
	Costs to deliver the BeUpstanding program	Program completion survey; implementation check
	Program satisfaction and perceived impact (end program only) for champions and staff	Follow-up staff survey, program completion survey, implementation check, staff focus groups^a^
**Maintenance**		
	Self-reported workplace sitting time collected 9-months after end-of program	Staff maintenance survey
	Use of activity policies and practices	Staff maintenance survey, champion interviews

^a^In a subsample only.

#### Adoption

*Work team characteristics* to be measured include organizational size, workplace location (postcode), industry, and team size. Team size is asked initially on the registration survey and confirmed by the project management team. Team size is visibly displayed on the feedback reports (staff surveys reports, performance completion reports) for champions, and champions have the opportunity to modify their team size within their individual profile page. To assess eligibility and inform accrual targets, information on sector, job roles, and proportion of the team undertaking desk-based work will also be assessed. To understand the health and well-being culture of the work team, champions will be asked if their team is currently participating in any other workplace wellness/health promotion programs, the everyday interest of the team in health and well-being (1=nonexistent, no one interested, to 5=very high, all/nearly all interested), the team’s motivation to sit less and move more at work (1=nonexistent, no one motivated, to 5=very high, all/nearly all motivated), and their team’s level of stress (1=minimal/no stress to 5=severe stress). Workplace readiness for change will be assessed via the context, change efficacy, and change-related effort subscales of the Workplace Readiness Questionnaire [51]. The workplace audit, which was adapted from the Checklist of Health Promotion Environments at Worksites [52], will be used to capture information on office layout, availability of height-adjustable desks, the physical environment (eg, access to public transport and centrally located bins), and the cultural/policy environment (eg, flexible work options).

*Champion characteristics* to be measured include sex; age (years), job classification (employee, team leader/middle management, and senior management/executive), and job title (open ended). Champions will also be asked if they have a Health and Safety role in their workplace, whether they have done any training in workplace health programs before, and whether they have delivered and/or evaluated a workplace health program before, with responses of yes, no, and unsure for each item. Champions will be asked what they hope to achieve with the program, an also to describe their current workplace culture in terms of sitting, standing, and moving (including any potential barriers and enablers to change).

#### Reach

The extent of participation of staff in the various BeUpstanding activities will be determined from the champion-reported team size, and champion-reported numbers or percentages participating in BeUpstanding events (eg, well-being committees, staff information workshop, launch party). Staff characteristics to be collected via the staff survey include age, sex, education, job classification, work hours, and the number of days in the last week where they had done a total of 30 min or more of physical activity, which was enough to raise their breathing rate [53]. Staff will also have the option to enter data about their postschooling education qualifications, whether they speak a language other than English at home, home postcode, height (cm), weight (kg), smoking status, and the number of times per week they usually did vigorous activity, walking, and other moderate-intensity activity [54]. The size and characteristics of teams taking part compared with the broader organization will be compared using champion-reported data collected via sign on and the Champion Profile Survey.

#### Implementation

The primary implementation outcome is program completion. At a minimum, successful completion is considered as completing all the core elements of the program (Table 1). Secondary implementation outcomes are engagement with the program (assessed through, eg, the number of log-ons to the toolkit, duration of using the toolkit, duration of running the program, and use of program materials), barriers and enablers to implementation, and costs of implementation (including time taken by the champion to plan, deliver, and evaluate the program, including gaining management support; see economic evaluation). Strategies chosen by the work team to BeUpstanding will be considered at a basic descriptive level (number of strategies chosen, frequency of certain strategies chosen) and according to the hierarchy of control (Table 2). Other factors tracked will include adaptions made (and desired) to the program materials by the work teams and participation by champions in activities to support engagement/implementation (eg, workshops for champions, champion forums).

#### Effectiveness

##### Workplace Sitting and Activity

The primary effectiveness outcome is self-reported workplace sitting time. This will be measured by the Occupational Sitting and Physical Activity Questionnaire (OSPAQ) [55], which asks about the percentage of time on a typical workday in the last 7 days spent sitting, standing, walking, and/or in heavy labor or physically demanding tasks. As such, it will also capture key secondary activity outcomes concerning time spent in other active behaviors at work: standing, walking, heavy labor, and moving (ie, walking + heavy labor). Measures from the OSPAQ have acceptable reliability and validity against posture-based activity monitors [56] and are responsive to change [56]. Participants will also be asked to estimate how many breaks from sitting they typically took in each hour while at work (six response options from 0 to 5 or more [57]) and the percentage of their sitting time at work they think is accrued in prolonged, unbroken, continuous bouts of 30 min or more (whole percentage from 0 to 100). This latter question was developed for the BeUpstanding study to capture change in prolonged sitting time. Unpublished testing within one of the early adopting workplaces (a call center; n=28 participants) showed acceptable test-retest reliability (*r*=0.74, 95% CI 0.51 to 0.87) and criterion validity (*r*=0.54, 95% CI 0.20 to 0.76) against workplace sitting in bouts of 30 min or more as recorded by the activPAL3 [58].

##### Activity Preference Alignment

Participants will be asked “if you were given a choice at work, what percentage of the time would you want to spend: sitting, standing, moving.” Activity preference alignment at work will be calculated as the absolute value of the difference between their preferred behavior and their self-reported behavior. The alignment scores for sitting, standing, and moving each theoretically range from 0 (desired and performed are exactly the same) to 100 (desiring 100% and doing 0% or vice versa) [36].

##### Organizational Social Norms

In line with the measure used in the pilot study [36], staff will be asked on a 5-point Likert scale (1=strongly disagree to 5=strongly agree) the extent to which they agree or disagree with five statements regarding control of how much they sit and stand at work; how much their organization is committed to supporting staff choices to sit, stand and move at work; whether management is supportive if they want to stand and move more at work; whether management *walks the talk* when it comes to modeling standing and moving more at work; and whether their work team has a culture that supports standing and moving. These five items will be used to create an *organizational social norms* score.

##### Enablers to Sitting Less and Moving More

Staff will be asked (yes/no) whether they believe that too much sitting is detrimental to their health and well-being, whether a dynamic work environment is beneficial to their productivity, whether they want to sit less at work, and whether they have access to a height-adjustable desk. These four items will be used to create an *enablers score*.

##### Perceived Barriers to Sitting Less and Moving More

Participants will be asked on a 5-point Likert scale (1=strongly disagree to 5=strongly agree) the extent to which they agree or disagree with seven statements regarding perceived barriers to sitting less and moving more at work: I am too busy to sit less at work, I worry that I would be perceived as being unproductive if I sat less at work, I need new equipment (eg, desk or headphones) to support me to sit less at work, the tasks I have to do in my job prevent me from being able to sit less at work, I worry that I would be perceived as *weird* if I sat less at work, my health prevents me from standing and moving more at work, and I need prompting to remember to sit less at work. Scores from these items will be used to create a *barriers score*. Participants will also be asked an open-ended question on any other factors that are preventing them from being able to sit, stand, or move at their desired levels at work.

##### Use of Activity-Promoting Strategies

Participants will be provided with a menu of common strategies that have been used to promote standing up, sitting less, and moving more in the desk-based environment inclusive of those promoted in the BeUpstanding resources [15,18,59] and will be asked on a 5-point Likert scale to indicate the extent to which they used these strategies (never, rarely, sometimes, often, very often/always, and not applicable). Scores from these items will be used to create a *strategy use score*.

##### Work Performance Indicators

Self-rated job performance [60] and job satisfaction [61] will be measured using single-item 7-point Likert scales. Participants will also be asked to rate on a 5-point scale (1=not at all to 5=extremely) the extent in the last week at work that they felt productive, creative, and part of a team. They will also be asked the number of days in the last 4 weeks (0-28 days) that they have stayed away from work for more than half the day because of health problems [62].

##### Perceived Health Status

Musculoskeletal symptoms in the last week will be measured using 3-items adapted from the Nordic Musculoskeletal Questionnaire [63,64] to assess the level of discomfort in (1) upper back, neck, shoulders, elbows, wrists, or hands; (2) lower back; and (3) hips, thighs, buttocks, knees, ankles, or feet. Each item will be assessed on an 11-point scale, from 0 (no discomfort at all) to 10 (severe discomfort). Current physical and mental health will each be rated on a single 5-point scale (1=poor to 5=excellent) [65,66]. To provide an indication of current stress and energy levels, participants will also be asked to rate on a 5-point scale (1=not at all to 5=extremely) the extent in the last week at work that they felt stressed, alert, energetic, and creative.

##### Adverse Events

The experience of any adverse events associated with program participation will be asked of both champions and staff.

##### Program Satisfaction and Feedback

Feedback on the BeUpstanding program will be sought from both champions and staff using fixed-option questions and qualitatively via open ended questions and qualitative interviews (in a subsample). Questions will cover program awareness, enjoyment, satisfaction, and potential for improvement. At the end of program, the staff survey will gather staff perceptions of the impact of the BeUpstanding program (negative impact, no/minimal impact, or positive impact) on five success dimensions: the culture in their work team around sitting, standing, and moving; their knowledge of the benefits of sitting less; their attitudes toward sitting, standing, and moving; their awareness of their sitting behavior; and their activity outside of work. Champions will be asked to report, using a 5-point Likert scale (1=not at all to 5=complete success), their perception of the extent to which the program raised awareness of the benefits of sitting less in the team, built a culture in their work team that supports sitting less and moving more, and reduced the amount their team engaged in prolonged unbroken sitting time. Adaptions and modifications to the program or program resources by the champions will be collected and recorded through the scheduled implementation checks.

#### Maintenance—Understanding Sustainability

At postprogram assessment (approximately 9 months after the 8-week program completion), champions will be interviewed to understand current workplace policies and practices related to sitting less and moving more and ongoing or new BeUpstanding strategy use. All staff will be sent the maintenance survey (a repeat of the baseline staff survey) to understand the sustainability of any changes.

#### Economic Evaluation

The economic evaluation will address the costs and outcomes of scaling up to national implementation, including intervention affordability and sustainability. The economic analysis will be undertaken from a societal perspective, but with the major focus on a workplace perspective (covering both costs and benefits to employers and employees). The study design lends itself to a cost-outcome description as a full economic evaluation such as cost-effectiveness analysis would require a control arm. The primary economic analysis will comprise the analyses of costs, outcomes, and the relationship between costs and outcomes. Detailed pathway analysis will be used to identify all resource use associated with the intervention delivery. The intervention will be assumed to be operating in steady state (ie, up and running at its full effectiveness potential), all costs associated with preplanning and development will be excluded. The included costs will relate to workplace recruitment (promotion events, social media, newsletters, etc) and intervention delivery (such as the staff workshop, posters, conduct of toolkit components, champion time, meetings of staff well-being committees, maintenance of website, etc). Data on the strategies adopted by individual work teams (including estimated costs) will be collected via the implementation checks. All resources will be valued in Australian dollars for the 2019 reference year. The economic outcomes for the implementation study will be presented as total costs, average costs per work team, and per work team of different size. Analysis of who incurs the associated costs (government, employers, individual employees, and research team) will be undertaken to assess intervention affordability and sustainability.

#### Data Analyses

Adoption, reach, and implementation outcomes will be described overall and within each priority sector. Effectiveness outcomes will also be evaluated overall and within each priority sector, with all work teams that are located in multiple sectors (eg, regional and small businesses) examined as part of every sector to which they belong. Effectiveness outcomes collected at the end of program only from champions and/or staff (eg, satisfaction) will be described. Effectiveness of the intervention on the primary outcome and secondary outcomes (continuous) collected repeatedly in the staff surveys will be assessed using mixed models that account for nonindependence in the form of individuals with repeated observations (baseline, end of program, and postprogram) and *team* clustering. The primary endpoint is the end of program (approximately 8 weeks). The pragmatic aspects of the champion-led collection of anonymous data from staff within a workplace means the staff surveys will be sent out to all staff who are team members at the time in a repeated cross-sectional fashion. Most are likely a core cohort sent all surveys (not known to the research team) who may respond to none or any number of the three surveys. In addition, some team members will be added or lost with workforce turnover. Accordingly, the evaluation will consist of assessing both changes within baseline responders who are followed up over time, and as this may be a select motivated subset, also assessing time trends in all evaluable cases (responders to any survey). Time trends will be considered both unadjusted and adjusting for potential compositional differences between responders at each assessment (because of variations in team membership with workforce turnover and who responds to each survey). To evaluate sensitivity of conclusions to missing data handling, multiple imputation analyses will also be performed. Team-level variation in effectiveness will be considered. If applicable, then program engagement, characteristics of the work teams and workplace champions, and the timing (month/year) of the intervention will be explored as reasons for the differential effectiveness.

Qualitative data from the focus groups with staff (effectiveness—barriers, enablers, and satisfaction) and semistructured interviews with champions (maintenance—use of policies and practices) will be audio-recorded and transcribed verbatim. Data from focus groups and champion interviews will be analyzed separately. Consistent with the recognized guidelines for qualitative data analyses [67], two members of the research team will independently code each transcript, where deductive codes will be identified based on the a priori constructs of interest (barriers, enablers, and satisfaction). Furthermore, all transcripts will be read to look for emergent themes (inductive coding). Initial codes will be grouped together into subthemes and overarching themes and relevant data to each theme collated. The coding frameworks developed by the research team members will then be compared for similarities or differences. Any discrepancies will be discussed with at least one other team member for consensus of the coding framework.

#### Sample Size for Primary Effectiveness Outcome

For the primary effectiveness outcome (work sitting), the minimum difference of interest will be 20 min per 8 hours at work, which is equivalent to two-thirds of the effect in the pilot (30 min/8 hours) [36] and what we might expect to see maintained in the long term [6]. Calculations using the GLIMMPSE software (version 2.2.8) indicate the study requires 47 to 62 teams to detect a change of this magnitude with 80% to 90% power and 5% two-tailed significance. Calculations assume, based on the pilot and early BeUpstanding data, an average of five workers per team will provide data (after attrition): SD 90, r=0.5, and intracluster correlation=0.1. Thus, to provide an adequate sample size to test effectiveness within every priority sector and overall, at least 50 work teams per priority sector will be recruited, with no fixed upper limit to recruitment within these priority sectors or other sectors.

## Results

Funding for the trial was obtained from June 1, 2018, to May 31, 2021. The protocol for the data collection was originally approved by the institutional review board on January 9, 2017, with the national implementation trial consent and protocol amendment approved on March 12, 2019. The start date for the trial was June 12, 2019. As of December 2019, 48 teams have been recruited into the trial.

## Discussion

Desk-based workers spend on average an estimated 70% to 80% of their workday sitting [6], putting their present and future health and productivity at risk. This novel implementation trial in work teams of desk-based workers across Australia will determine whether the BeUpstanding Champion Toolkit is a feasible, effective, safe, and economical resource for sustainably reducing workplace sitting. The multilevel and mixed method evaluation will also enable examination of the predictors of success across a wide range of employment sectors, including sectors that have been underserved and underresearched. Through explicit consideration of a wide range of potential benefits and possible adverse events, it should be possible in the future to provide many of the answers to questions and concerns that could arise during more widespread adoption. Findings will provide the fundamental practice-based evidence needed to inform workplace health, policy, and practice on effective and sustainable ways to promote more movement and less sitting without compromising productivity or worker health. These practice-based findings will also inform the potential for broader dissemination of the toolkit, providing an opportunity to advance the translational evidence base. Importantly, as the program is freely available with no upper limit to enrolment, there is the opportunity to compare outcomes and engagement of those recruited into the implementation trial compared with those participating in the BeUpstanding program but not taking part in the trial.

### Limitations and Strengths

As an implementation study, there are some inherent limitations. The use of a single-group, pre- to poststudy design is primary among these. A randomized controlled trial (RCT) design was considered, as this design would provide more robust effectiveness outcomes. However, an RCT would not provide better data for the reach, adoption, and implementation outcomes. It was also unclear how to conduct an RCT while preserving the key intervention model being tested of a workplace champion delivering and evaluating the intervention, particularly given the BeUpstanding toolkit is already live and freely available. Experience from the pilot and early adopters phases (phases 2 and 3) led us to expect that we would not be able to recruit champions willing to act as controls and complete all the evaluation but receive none of the intervention (even if they received a delayed intervention). Even the evaluation requires a reasonable amount of effort on the part of the workplace champion: researchers have no contact with the staff. Anyone can sign up to the toolkit (including potential control organizations) meaning contamination would be very difficult to control in those who sign up and are allocated to the control arm. We would also need to expend significant resources tailoring the toolkit to perform the evaluation but not the delivery intervention functions for those champions whose teams were allocated to a control condition. Therefore, on balance, it was considered that the pre-post design was the most appropriate to evaluate the implementation trial.

Providing a menu of options and supporting work teams to collectively choose which intervention strategies will work best for them is a key strength of the program, with findings likely to provide key insights into possible higher order strategies to effectively support workers to sit less and move more [68], but this approach does mean that findings across work teams will not necessarily be directly comparable. It also means that strategies known to successfully achieve shifts in workplace sitting time, such as the use of sit-stand workstations as part of a multicomponent approach [35], will not necessarily be implemented by work teams. Furthermore, for some individuals, the strategies chosen by the team to BeUpstanding may not be appropriate for them personally. However, the primary questions to be answered are about the uptake, implementation, and costs of wide-scale implementation and the outcomes that can be achieved in this context; questions that are being answered through RE-AIM—a widely used framework for understanding dissemination [49]. Further strengths of the study include its pragmatic design. The toolkit readily facilitates uptake and delivery with minimal follow-up required from stakeholders. The program is also designed to be easily integrated into existing wellness, health, and safety initiatives. This presents an innovative model that has a high likelihood of being able to be generalized more broadly. Importantly, all five industry partners are ideally suited to use trial findings to directly shape and deliver national and international workplace policy and practice.

## Appendix

Multimedia Appendix 1 CONSORT-eHEALTH checklist (V 1.6.1).

Multimedia Appendix 2 Application assessment summary from Australian National Health and Medical Research Council (NHMRC).

## Abbreviations

NHMRC: National Health and Medical Research Council
OSPAQ: Occupational Sitting and Physical Activity Questionnaire
RCT: randomized controlled trial
