# Supplementary material for: Supporting Workers to Sit Less and Move More Through the Web-Based BeUpstanding Program: Protocol for a Single-Arm, Repeated Measures Implementation Study
Source: JMIR Res Protoc. 2020 May 4;9(5):e15756. doi: 10.2196/15756 (PMC7235812; doi:10.2196/15756)
Supplement: Multimedia Appendix 2 [file resprot_v9i5e15756_app2.doc]

**APP1149936 Application Assessment Summary**

Your Partnership Project 2017 First Call application was scored in **Category 5** following its assessment by the Partnership Projects Peer Review Panel (PRP).

**Table 1:** Summary of the assessment of your application against the Partnership Projects Assessment Criteria.

| **Criteria** | **Scores for APP1149936** |
| --- | --- |
| 1- Track Records of the Chief Investigators Relative to Opportunity (25%) | 5.778 |
| 2- Scientific Quality of the Proposal and Methodology (25%) | 4.000 |
| 3- Relevance and Likelihood to Influence Health and Research Policy and Practice (25%) | 4.111 |
| 4- Strength of Partnership (25%) | 4.556 |
| **Overall Category** | **5** |

**Table 2:** The proportion of Partnership Projects applications in each category. This table includes all Partnership Projects applications for this call that were fully assessed by the PRP (i.e. it does not include applications deemed ‘Not For Further Consideration’ after initial assessment). Mean scores (± 1 standard deviation) for each criterion are provided for each category.

| **Category** | **Number and Proportion (%) of applications in Category** | **Track Records of CIs Relative to Opportunity**  **(mean)** | **Scientific Quality of the Proposal and Methodology**  **(mean)** | **Relevance and Likelihood to Influence Health and Research Policy and Practice**  **(mean)** | **Strength of Partnership**  **(mean)** |
| --- | --- | --- | --- | --- | --- |
| 7 | Nil | Nil | Nil | Nil | Nil |
| 6 | 2 (10%) | 5.900 ± 0.141 | 5.200 ± 0 | 5.800 ± 0.283 | 5.700 ± 0.141 |
| 5 | 11 (55%) | 5.488 ± 0.247 | 4.704± 0.610 | 4.794 ± 0.532 | 5.091 ± 0.501 |
| 4 | 6 (30%) | 4.697 ± 0.312 | 4.020 ± 0.195 | 3.969 ± 0.116 | 4.304 ± 0.304 |
| ≤3 | 1 (5%) | 3.400 ± 0 | 2.800 ± 0 | 3.000 ± 0 | 3.200 ± 0 |

Panels comments (as provided by the Primary Spokesperson)

**1 Track records of the Chief Investigators relative to opportunity**

This is a very strong group of researchers. The panel raised concern that given the technical nature of the study it would be good to have a CI involved who has knowledge/capacity with programming/web design. It was suggested that it is a very large undertaking and it is unclear that the CI’s have experience successfully implementing a website of this scale. The CIs have strong track records of continuous funding for many years. It is not very clear how many of these grants remain current and, therefore, the teams’ capacity to complete the proposed work.

**2 Scientific quality of the proposal and methodology**

The panel raised significant concerns about the importance of changing sitting time with respect to cardiovascular health considering the growing body of evidence questioning its importance. The CI’s own small RCT showed small benefits in only some biomarkers and only if a 12 month intervention was performed. It is unclear why a focus on sitting is important when improving physical activity levels would have a far greater impact and seems just as achievable. The panel was concerned that this would be a very large disruption to the workforce for only minimal potential benefits. The panel raised the issue that the secondary outcomes (productivity, job satisfaction, musculoskeletal symptoms etc.) seem at least as important as the primary ones but were not described in detail in the application. Issues were raised about the safety of the intervention in older aged workers related to falls and joint pain. The data collection and intervention implementation is reliant on the participants themselves. Whilst the Project manager will follow up with reminders via email/phone/SMS this represents a risk. Stronger evidence of the implementation science used to engage the champions could have been presented. Additionally, the detail provided about the intervention is very broad.

**3 Relevance and likelihood to influence health and research policy**

The panel stated that it was unclear how relevant sitting behaviour change is to improving long term cardiovascular health outcomes. This is particularly important relevant to other aspects which could result in far greater improvements (eg. a single session of moderate to high intensity activity per week). There is no doubt that standing desks etc. are becoming more prevalent and there is an opportunity to change practice, however whether this is warranted is unknown. Student projects are said to be embedded within the proposal, however, this is not clear how this will be achieved. There is somewhat less convincing evidence of the capacity mix within the CI team (CI Goode listed as ECR but PhD in 2012 and CI on another NHMRC).

**4 Strength of the partnership**

This looks like a very strong partnership between elite universities/research centres and external bodies. There does seem to be strong evidence of linkage between all the groups involved. However not enough cash was contributed given the size of the companies involved.
